# Association of early feeding patterns with physical growth and neurodevelopment in term high-risk infants: a retrospective study

**DOI:** 10.3389/fpubh.2026.1995315

**Published:** 2026-10-28

**Authors:** Cheng-Cheng Zhang, Xiao-Jing Xu, Yan Li, Ying-Yan Wang

**Affiliations:** Department of Child Health Care, Affiliated Maternity and Child Health Care Hospital of Nantong University, Nantong, Jiangsu, China

**Keywords:** breastfeeding, feeding patterns, neurodevelopment, physical growth, term high-risk infants

## Abstract

**Background and objectives:**

The relationship between early feeding patterns and growth and neurodevelopment in term high-risk infants remains unclear. This study aimed to investigate the associations between feeding patterns during the first 6 months of life and physical growth and neurodevelopment in term high-risk infants.

**Subjects and methods:**

A total of 108 term high-risk infants were retrospectively included and classified according to feeding patterns during the first 6 months of life into an exclusive breastfeeding group (*n* = 32), a mixed feeding group (*n* = 45), and a formula feeding group (*n* = 31). Data on weight, length, and head circumference at 6, 8, and 12 months were collected. Weight-for-age *Z* score (WAZ), length-for-age *Z* score (LAZ), and head circumference-for-age *Z* score (HCZ) were calculated using WHO Anthro software. Neurodevelopment at 8 and 12 months was assessed using the Developmental Screening Test (DST) for children aged 0–6 years in China. Linear mixed-effects models were used to analyze the associations between feeding patterns and physical growth trajectories, with adjustment for sex, birth weight, fetal/neonatal risk factors, and maternal risk factors.

**Results:**

In unadjusted analyses, the exclusive breastfeeding group had higher WAZ and LAZ at 6 months, higher WAZ and HCZ at 8 months, and higher WAZ at 12 months than the formula feeding group (all *p* < 0.05). After adjustment for potential confounders, the feeding group × time interaction was significant for LAZ (*p* = 0.004), but not for WAZ (*p* = 0.265) or HCZ (*p* = 0.168). At 6 months, WAZ (adjusted MD = 0.721, 95% CI: 0.126–1.316; *p* = 0.012) and LAZ (adjusted MD = 0.602, 95% CI: 0.059–1.144; *p* = 0.024) remained higher in the exclusive breastfeeding group than in the formula feeding group, whereas no significant between-group differences were observed at 8 or 12 months. No significant differences in DQ or developmental classification were observed among the feeding groups at 8 or 12 months (all *p* > 0.05).

**Conclusion:**

Early feeding patterns were associated with growth trajectories in term high-risk infants, and breastfeeding may have been associated with more favorable physical growth in early life. No clear differences in neurodevelopmental outcomes were observed among feeding patterns; however, these findings should be interpreted cautiously because of the limited sample size available for neurodevelopmental assessment.

## 1 Introduction

High-risk infants are defined as infants exposed to biological, maternal, or psychosocial environmental risk factors before or around birth, resulting in an increased risk of impaired growth, developmental delay, and adverse health outcomes (1). Adverse perinatal exposures, including fetal growth restriction, perinatal hypoxia, and perinatal infection, may adversely affect fetal and brain development and are associated with increased risks of impaired growth, neurodevelopmental disorders, and subsequent cognitive or behavioral abnormalities (2, 3). To improve long-term outcomes among high-risk infants, China has established a specialized healthcare management system incorporating standardized follow-up, developmental surveillance, and early intervention programs (1).

The World Health Organization (WHO) recommends exclusive breastfeeding for the first 6 months of life (4). When exclusive breastfeeding is not feasible because of medical, maternal, or practical circumstances, mixed feeding or formula feeding may also be used according to individual needs and circumstances. Previous studies have reported associations between feeding patterns and infant growth or neurodevelopment, but the populations studied and outcomes assessed have varied considerably (5–7).

Much of the available evidence on feeding and growth has been derived from generally healthy infant populations (5, 8), whereas studies in high-risk populations have often focused on preterm or low-birth-weight infants (7). Term high-risk infants may have distinct growth and neurodevelopmental trajectories because of underlying perinatal risk factors, and evidence specifically addressing the associations between feeding patterns and developmental outcomes in this population remains limited. Therefore, this retrospective study was conducted to investigate the associations between different feeding practices during the first 6 months of life and physical growth and neurodevelopment among term high-risk infants, aiming to provide evidence for early feeding management and developmental surveillance in this vulnerable population.

## 2 Materials and methods

### 2.1 Study population and ethics

This retrospective study included 108 term high-risk infants who underwent regular follow-up at the High-Risk Infant Clinic, Department of Child Health Care, Affiliated Maternity and Child Health Care Hospital of Nantong University, between February 2024 and February 2026. According to the Chinese Expert Consensus on Standardized Health Management for High-Risk Infants (1), high-risk infants are defined as children who, from the fetal period to 3 years of age, have biological, psychological, social, environmental, or other risk factors that may affect their physical or mental development.

The inclusion criteria were as follows: (1) meeting the criteria for high-risk infants (1); (2) gestational age ≥37 weeks; and (3) birth weight >1,500 g. The exclusion criteria were: (1) incomplete clinical data; (2) congenital malformations or inherited metabolic disorders; and (3) failure to complete the scheduled follow-up. Thus, the study population represented a subset of high-risk infants who were born at term.

The high-risk conditions documented in the electronic medical records of the included infants comprised low birth weight, fetal distress, neonatal hyperbilirubinemia, neonatal hypoglycemia, neonatal pneumonia, neonatal sepsis, gestational diabetes mellitus, hypertensive disorders of pregnancy, maternal hypothyroidism, and advanced maternal age. These conditions were extracted directly from the existing clinical records and were not retrospectively re-diagnosed or reclassified by the investigators.

This study was approved by the Ethics Committee of the Affiliated Maternity and Child Health Care Hospital of Nantong University (Approval No. Y2021059). Written informed consent was waived due to the retrospective nature of this study.

Feeding patterns during the first 6 months were determined retrospectively from feeding histories documented at routine follow-up visits. Infants who received breast milk without formula supplementation during this period were classified into the exclusive breastfeeding group; those who received both breast milk and formula were classified into the mixed feeding group; and those who received formula without breast milk were classified into the formula feeding group. Partial breastfeeding was included in the mixed feeding group. Complementary foods were introduced at approximately 6 months of age and therefore did not affect the classification of feeding patterns during the first 6 months (Figure 1).

**Figure 1:**
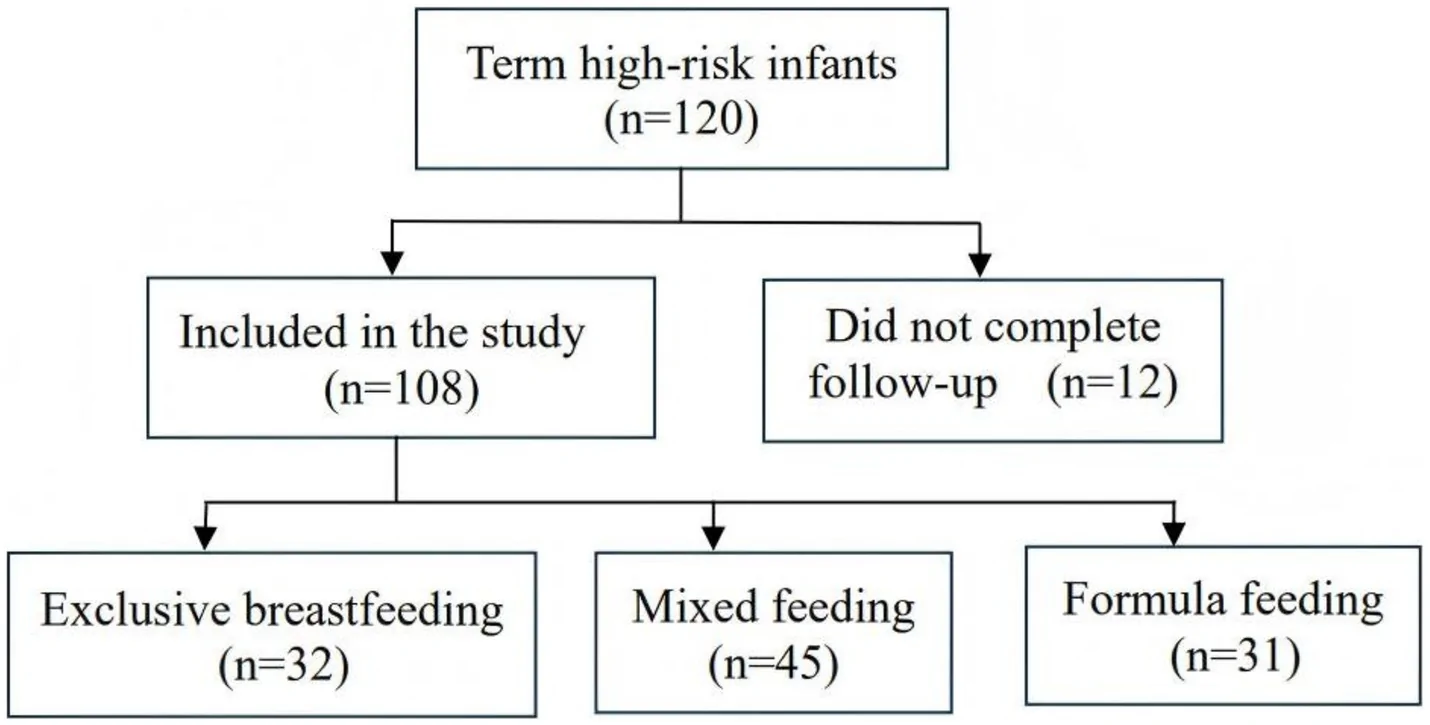
Flowchart of participant selection and grouping.

### 2.2 Follow-up and outcome assessment

All infants were followed for at least 12 months. Follow-up visits were scheduled every 1–2 months during the first 6 months of life, every 2–3 months between 7 and 12 months, and every 3–6 months thereafter. Body weight, length, and head circumference were measured at each follow-up visit.

Sex- and age-specific anthropometric *Z* scores, including weight-for-age *Z* score (WAZ), length-for-age Z score (LAZ), and head circumference-for-age *Z* score (HCZ), were calculated using WHO Anthro software based on the WHO Child Growth Standards. Neuropsychological development was assessed by certified physicians who had received standardized training using the Developmental Screening Test (DST) for children aged 0–6 years in China (9). Developmental outcomes were expressed as the developmental quotient (DQ) or mental index (MI). According to the DST criteria, DQ or MI ≥ 85 indicated normal development, 70–84 indicated suspected developmental delay, and <70 indicated developmental delay. For children younger than 3 years, only the DQ was used to evaluate neurodevelopment.

### 2.3 Data collection

Clinical data were retrospectively extracted from the hospital electronic medical record system, including demographic and perinatal characteristics (sex, birth weight, mode of delivery, gestational age, high-risk factors, and feeding pattern), anthropometric measurements (body weight, length, and head circumference) at 6, 8, and 12 months of age, and neuropsychological assessment results at 8 and/or 12 months of age. For some infants, gestational age was documented only as “>37 weeks” rather than as an exact value. These records were retained as originally documented, without assigning an estimated gestational age. All data were entered into Microsoft Excel by two independent investigators and were repeatedly cross-checked to ensure data accuracy and completeness.

### 2.4 Statistical analysis

Statistical analyses were performed using IBM SPSS Statistics version 26.0 (IBM Corp., Armonk, NY, USA). The normality of continuous variables was assessed using the Shapiro–Wilk test. Normally distributed continuous variables were expressed as mean ± standard deviation and compared among groups using one-way analysis of variance (ANOVA), whereas non-normally distributed variables were expressed as median (P25, P75) and compared using the Kruskal–Wallis H test. When the overall between-group difference was statistically significant, *post hoc* pairwise comparisons were performed with Bonferroni correction. Categorical variables were expressed as *n* (%) and compared using the chi-square test or Fisher’s exact test, as appropriate.

To account for repeated measurements and potential confounding, separate linear mixed-effects models were fitted for WAZ, LAZ, and HCZ using maximum likelihood (ML) estimation. Feeding group, time (6, 8, and 12 months), and the feeding group × time interaction were included as fixed effects, with a subject-specific random intercept. All available repeated growth measurements were used in the models without imputation of missing outcome values, under the missing-at-random assumption. Three infants with missing birth weight data were excluded from the adjusted models. Sex, birth weight, fetal/neonatal risk factors, maternal risk factors, and advanced maternal age were included as covariates. Fetal/neonatal and maternal risk factors were each coded as binary composite variables indicating the presence of at least one corresponding risk condition. The fetal/neonatal risk composite variable included neonatal hyperbilirubinemia, hypoglycemia, pneumonia, sepsis, and fetal distress. The maternal risk composite variable included gestational diabetes mellitus, hypertensive disorders of pregnancy, and maternal hypothyroidism. Low birth weight and advanced maternal age were excluded from the respective composite variables because the corresponding variables were entered separately into the models. Model-based pairwise comparisons between feeding groups were adjusted using the Bonferroni method. All tests were two-sided, and *p* < 0.05 was considered statistically significant.

## 3 Results

### 3.1 Clinical characteristics of term high-risk infants

A total of 120 term high-risk infants were initially enrolled in this study. Among them, 12 infants who failed to complete the follow-up were excluded, and a final sample of 108 infants was included in the subsequent statistical analysis, comprising 54 males and 54 females (Table 1). The infants were classified into the exclusive breastfeeding group (*n* = 32), mixed feeding group (*n* = 45), and formula feeding group (*n* = 31) according to feeding exposure during the first 6 months of life (Figure 1).

**Table 1:** Comparison of demographic characteristics and perinatal risk factors among groups of high-risk infants.

Characteristics	Exclusive breastfeeding (*n* = 32)	Mixed feeding (*n* = 45)	Formula feeding (*n* = 31)	*χ*^2^/*F*	*p* value
Sex					
Female	47% (15/32)	53% (24/45)	48% (15/31)	0.357	0.836
Male	53% (17/32)	47% (21/45)	52% (16/31)		
Mode of delivery					
Vaginal delivery	58% (15/26)	52% (17/33)	39% (9/23)	1.733	0.420
Caesarean	42% (11/26)	48% (16/33)	61% (14/23)		
Birth weight (g)	3421.25 ± 451.10, *n* = 32	3207.11 ± 529.65, *n* = 45	3093.82 ± 594.85, *n* = 28	3.070	0.051
Risk factors for high-risk infants					
Hyperbilirubinemia	13% (4/32)	11% (5/45)	16% (5/31)		
Low birth weight	6% (2/32)	13% (6/45)	13% (4/31)		
Fetal distress	13% (4/32)	9% (4/45)	3% (1/31)		
Neonatal hypoglycemia	3% (1/32)	4% (2/45)	0% (0/31)		
Neonatal pneumonia	3% (1/32)	0% (0/45)	6% (2/31)		
Neonatal sepsis	3% (1/32)	0% (0/45)	0% (0/31)		
Maternal GDM	47% (15/32)	47% (21/45)	48% (15/31)		
Maternal HDP	22% (7/32)	18% (8/45)	16% (5/31)		
Maternal hypothyroidism	9% (3/32)	2% (1/45)	13% (4/31)		
Advanced maternal age	9% (3/32)	4% (2/45)	23% (7/31)		

Data were presented as percentages (*n*/*N*) or mean ± standard deviation (SD), as appropriate. Percentages were calculated using the number of infants with available data for each variable as the denominator. Missing data were observed for mode of delivery (*n* = 26) and birth weight (*n* = 3). Specifically, mode of delivery data were missing for 6, 12, and 8 infants in the exclusive breastfeeding, mixed feeding, and formula feeding groups, respectively; birth weight data were missing for 3 infants in the formula feeding group.

Some infants had more than one perinatal risk factor; therefore, the risk factors were not mutually exclusive, and their percentages could sum to more than 100%. Perinatal risk factors were presented descriptively without statistical comparisons because of the small number of events in some categories.

GDM, gestational diabetes mellitus; HDP, hypertensive disorders of pregnancy. A two-sided *p* value <0.05 was considered statistically significant.

Some baseline variables had missing data. Mode of delivery data were available for 82 infants, while birth weight data were available for 105 infants. Among the 108 infants, exact gestational age was documented for 44 infants (41%), whereas gestational age was recorded only as “>37 weeks” for the remaining 64 infants (59%). All infants were documented as having been born at term (Supplementary Table S1). The major maternal risk factors included gestational diabetes mellitus, hypertensive disorders of pregnancy, advanced maternal age, and maternal hypothyroidism. The main fetal and neonatal risk factors included hyperbilirubinemia, low birth weight, and fetal distress. Some infants had more than one high-risk condition (Table 1 and Supplementary Table S1).

There were no statistically significant between-group differences in sex, mode of delivery, or birth weight (all *p* > 0.05). However, mean birth weight was numerically higher in the exclusive breastfeeding group than in the formula feeding group (3421.25 *vs* 3093.82 g), although the overall between-group comparison did not reach statistical significance (*p* = 0.051) (Table 1).

### 3.2 WHO-standardized anthropometric *Z* scores at 6, 8, and 12 months according to feeding pattern

At 6 months, significant between-group differences were observed in LAZ (*p* = 0.007) and WAZ (*p* = 0.003), whereas HCZ did not differ significantly among the three groups (*p* = 0.080) (Table 2 and Supplementary Table S3). After Bonferroni correction, the exclusive breastfeeding group had higher LAZ (MD = 0.747, 95% CI: 0.156–1.339; *p* = 0.008) and WAZ (MD = 0.924, 95% CI: 0.266–1.583; *p* = 0.003) than the formula feeding group; no other pairwise comparisons were statistically significant (Supplementary Table S2).

**Table 2:** Comparison of WHO-standardized anthropometric *Z* scores at 6, 8, and 12 months among term high-risk infants according to feeding pattern.

Age and growth indicator	Exclusive breastfeeding (*n* = 32)	Mixed feeding (*n* = 45)	Formula feeding (*n* = 31)	*F*	*p* value
6 months					
Length-for-age *z* score (LAZ)	1.06 ± 0.90, *n* = 32	0.52 ± 0.99, *n* = 45	0.31 ± 0.99, *n* = 31	5.161	0.007*
Weight-for-age *z* score (WAZ)	1.21 ± 0.97, *n* = 32	0.64 ± 1.17, *n* = 45	0.29 ± 1.04, *n* = 31	5.978	0.003*
Head circumference-for-age z score (HCZ)	0.60 ± 0.86, *n* = 32	0.27 ± 0.96, *n* = 45	0.06 ± 1.01, *n* = 31	2.591	0.080
8 months					
Length-for-age *z* score (LAZ)	0.92 ± 0.93, *n* = 25	0.34 ± 1.04, *n* = 29	0.28 ± 1.07, *n* = 26	3.148	0.049*
Weight-for-age *z* score (WAZ)	1.18 ± 0.94, *n* = 25	0.57 ± 1.13, *n* = 29	0.21 ± 1.05, *n* = 26	5.647	0.005*
Head circumference-for-age z score (HCZ)	0.68 ± 0.96, *n* = 25	0.09 ± 0.92, *n* = 29	−0.07 ± 1.01, *n* = 26	4.331	0.016*
12 months					
Length-for-age *z* score (LAZ)	0.52 ± 0.87, *n* = 32	0.35 ± 0.97, *n* = 44	0.21 ± 1.08, *n* = 29	0.793	0.455
Weight-for-age *z* score (WAZ)	0.84 ± 0.90, *n* = 32	0.43 ± 1.03, *n* = 44	0.16 ± 1.10, *n* = 29	3.470	0.035*
Head circumference-for-age *z* score (HCZ)	0.45 ± 0.97, *n* = 32	0.22 ± 0.81, *n* = 44	0.08 ± 1.03, *n* = 29	1.280	0.283

Values are presented as mean ± standard deviation. WAZ, weight-for-age *Z* score; LAZ, length-for-age *Z* score; HCZ, head circumference-for-age *Z* score. WAZ, LAZ, and HCZ were calculated using WHO Anthro software based on sex- and age-specific WHO Child Growth Standards. Differences among the three feeding groups were assessed using one-way analysis of variance. A two-sided P value <0.05 was considered statistically significant. *p < 0.05.

At 8 months, significant between-group differences were observed in LAZ (*p* = 0.049), WAZ (*p* = 0.005), and HCZ (*p* = 0.016) (Table 2 and Supplementary Table S4). After Bonferroni correction, the exclusive breastfeeding group had higher WAZ (MD = 0.975, 95% CI: 0.257–1.693; *p* = 0.004) and HCZ (MD = 0.752, 95% CI: 0.093–1.410; *p* = 0.020) than the formula feeding group, whereas pairwise comparisons for LAZ and all other comparisons were not statistically significant (Supplementary Table S2).

At 12 months, only WAZ differed significantly among the three groups (*p* = 0.035), whereas LAZ (*p* = 0.455) and HCZ (*p* = 0.283) did not (Table 2 and Supplementary Table S5). After Bonferroni correction, the exclusive breastfeeding group had higher WAZ than the formula feeding group (MD = 0.676, 95% CI: 0.042–1.310; *p* = 0.033), while no other pairwise comparisons were statistically significant (Supplementary Table S2).

### 3.3 Adjusted longitudinal analysis of anthropometric *Z* scores

In linear mixed-effects models adjusted for prespecified covariates, the feeding group × time interaction was significant for LAZ (*p* = 0.004), but not for WAZ (*p* = 0.265) or HCZ (*p* = 0.168) (Table 3). Bonferroni-adjusted pairwise comparisons showed that, at 6 months, the exclusive breastfeeding group had higher WAZ (adjusted MD = 0.721, 95% CI: 0.126–1.316; *p* = 0.012) and LAZ (adjusted MD = 0.602, 95% CI: 0.059–1.144; *p* = 0.024) than the formula feeding group. No other pairwise comparisons at 8 or 12 months were statistically significant (Table 3).

**Table 3:** Adjusted longitudinal mixed-effects analysis of growth *Z* scores according to feeding patterns.

Outcome	Age (months)	Exclusive breastfeeding *vs* formula feeding, adjusted mean difference (95% CI), Bonferroni-adjusted *p* value	Mixed feeding *vs* formula feeding, adjusted mean difference (95% CI), Bonferroni-adjusted *p* value	Exclusive breastfeeding *vs* mixed feeding, adjusted mean difference (95% CI), Bonferroni-adjusted *p* value	Feeding group × time interaction *p* value
WAZ	6	0.721 (0.126–1.316), *p* = 0.012*	0.328 (−0.220–0.877), *p* = 0.446	0.393 (−0.122–0.908), *p* = 0.199	0.265
	8	0.567 (−0.039–1.174), *p* = 0.075	0.237 (−0.325–0.800), *p* = 0.924	0.330 (−0.200–0.860), *p* = 0.399	
	12	0.480 (−0.116–1.076), *p* = 0.159	0.239 (−0.311–0.789), *p* = 0.882	0.241(−0.274–0.756), *p* = 0.774	
LAZ	6	0.602 (0.059–1.144), *p* = 0.024*	0.243 (−0.257–0.744), *p* = 0.722	0.358 (−0.112–0.828), *p* = 0.200	0.004*
	8	0.434 (−0.126–0.993), *p* = 0.187	0.182 (−0.339–0.703), *p* = 1.000	0.252 (−0.240–0.744), *p* = 0.652	
	12	0.156 (−0.389–0.700), *p* = 1.000	0.178 (−0.325–0.681), *p* = 1.000	−0.023 (−0.493–0.448), *p* = 1.000	
HCZ	6	0.241 (−0.279–0.761), *p* = 0.791	0.081 (−0.399–0.561), *p* = 1.000	0.160 (−0.291–0.610), *p* = 1.000	0.168
	8	0.444 (−0.093–0.981), *p* = 0.141	0.212 (−0.288–0.712), *p* = 0.919	0.232 (−0.241–0.705), *p* = 0.709	
	12	0.098 (−0.425–0.620), *p* = 1.000	0.031 (−0.451–0.514), *p* = 1.000	0.066 (−0.385–0.518), *p* = 1.000	

Values are presented as adjusted mean differences (95% confidence intervals). Linear mixed-effects models included feeding group, time, the feeding group × time interaction, sex, birth weight, fetal/neonatal risk factors, maternal risk factors, and advanced maternal age as fixed effects, with a subject-specific random intercept. The feeding group × time *p* value represents the overall interaction effect across all follow-up time points. Pairwise comparisons were adjusted using the Bonferroni method. Positive values indicate a higher *Z* score in the first-named feeding group relative to the second. WAZ, weight-for-age *Z* score; LAZ, length-for-age *Z* score; HCZ, head circumference-for-age *Z* score. *p < 0.05.

### 3.4 Comparison of neuropsychological development at 8 and 12 months of age

At 8 months, DQ data were available for 9, 17, and 15 infants in the exclusive breastfeeding, mixed feeding, and formula feeding groups, respectively; no significant between-group differences were observed in DQ or developmental classification (*p* = 0.314 and *p* = 0.728, respectively). At 12 months, the corresponding sample sizes were 12, 18, and 10, with no significant between-group differences in DQ or developmental classification (*p* = 0.838 and *p* = 0.889, respectively) (Table 4 and Supplementary Table S6).

**Table 4:** Comparison of developmental quotient outcomes at 8 and 12 months of age among high-risk term infants with different feeding patterns.

Variable	Exclusive breastfeeding (*n* = 32)	Mixed feeding (*n* = 45)	Formula feeding (*n* = 31)	*χ*^2^/F/H	*p* value
DST outcomes at 8 months					
DST developmental quotient classification, *n* (%)					0.728^#^
Normal	5 (56%)	5 (29%)	5 (33%)		
Suspected	4 (44%)	10 (59%)	8 (54%)		
Abnormal	0 (0%)	2 (12%)	2 (13%)		
DST developmental quotient	89 (78, 98), *n* = 9	83 (78, 88), *n* = 17	78 (72, 89), *n* = 15	2.319	0.314
DST outcomes at 12 months					
DST developmental quotient classification, *n* (%)				0.235	0.889
Normal	7 (58%)	9 (50%)	5 (50%)		
Suspected	5 (42%)	9 (50%)	5 (50%)		
Abnormal	0 (0%)	0 (0%)	0 (0%)		
DST developmental quotient	88 ± 15, *n* = 12	87 ± 10, *n* = 18	85 ± 7, *n* = 10	0.177	0.838

The number of participants varied because of incomplete DST assessments at each follow-up. ^**#**^*p* value obtained using Fisher’s exact test.

## 4 Discussion

This study evaluated the associations between different early feeding patterns and physical growth and neurodevelopment in term high-risk infants. The main finding was that exclusive breastfeeding during the first 6 months was associated with more favorable early growth patterns in term high-risk infants. Compared with formula-fed infants, exclusively breastfed infants had higher length-for-age and weight-for-age measures at 6 months, and these differences remained observable at 8 months, particularly in terms of weight gain and head circumference growth. After further adjustment for birth weight, sex, perinatal risk factors, and maternal factors, exclusive breastfeeding remained associated with higher weight-for-age and length-for-age Z-scores at 6 months. However, a significant feeding group × time interaction was observed only for LAZ, but not for WAZ or HCZ.

Human milk provides an appropriate balance of macronutrients required for infant growth and also contains a variety of bioactive components, including growth factors, hormones, human milk oligosaccharides, and lactoferrin. These components may support early growth through the provision of essential nutrients and modulation of intestinal development, gut microbiota, metabolism, and immune function (10, 11). For infants with high-risk conditions, these biological effects associated with breast milk might have been particularly relevant. The higher WAZ and LAZ observed in the exclusive breastfeeding group in this study are broadly consistent with previous studies reporting associations between breastfeeding and early physical growth (12–14).

Notably, the growth differences in this study were mainly observed between the exclusive breastfeeding and formula feeding groups, whereas no significant differences were found between the exclusive breastfeeding and mixed feeding groups. Similar differences in growth patterns among exclusively breastfed, partially breastfed, and formula-fed infants have been reported previously (13).

As follow-up progressed, the growth differences among feeding groups gradually diminished. Although the exclusive breastfeeding group had a higher WAZ than the formula feeding group at 12 months in the unadjusted analysis, this difference was no longer statistically significant after adjustment for potential confounders. These findings suggest that differences associated with early feeding patterns may persist into later infancy at the descriptive or unadjusted level, although the magnitude of these differences appears to diminish with age. After 6 months of age, complementary foods are gradually introduced and become increasingly important sources of energy and nutrients, and subsequent growth may therefore be influenced by complementary feeding and other feeding-related factors (15, 16). These changes may partly explain the attenuation of growth differences among feeding groups during later infancy.

With regard to neurodevelopment, no significant differences in developmental quotient or developmental classification were observed among the different feeding groups at either 8 or 12 months of age. Previous observational studies have suggested that breastfeeding duration may be associated with later cognitive and neurodevelopmental outcomes, although findings have varied across study populations, assessment tools, and developmental domains (17, 18). However, the relatively limited sample size available for neurodevelopmental assessment in this study might have reduced the ability to detect potential developmental differences. Therefore, larger, multicenter studies with long-term follow-up are needed to further clarify the potential associations between breastfeeding and neurodevelopment in term high-risk infants.

This study had several limitations. First, because of the retrospective study design, potential selection bias and residual confounding could not be completely excluded. Second, some factors associated with long-term child development, such as the amount of breast milk intake and the home-rearing environment, could not be further quantified. Future multicenter prospective studies with longer-term follow-up are needed to further investigate the associations between early feeding patterns and growth and developmental outcomes in term high-risk infants. Despite these limitations, this study incorporated follow-up assessments at multiple time points and analyzed the associations between different feeding patterns and growth outcomes after adjustment for relevant confounding factors, providing further evidence for early nutritional management in term high-risk infants.

## 5 Conclusion

Exclusive breastfeeding during the first 6 months of life was associated with more favorable early growth patterns among term high-risk infants. Because of the retrospective observational design, these findings should not be interpreted as evidence of a causal effect. No significant differences in neurodevelopment were observed among the different feeding groups; however, these findings should be interpreted cautiously given the limited sample size available for neurodevelopmental assessment.

## Data Availability

The raw data supporting the conclusions of this article will be made available by the authors, without undue reservation.
