# Supplementary material for: Association of early feeding patterns with physical growth and neurodevelopment in term high-risk infants: a retrospective study
Source: Front Public Health. 2026 Oct 28;14:1995315. doi: 10.3389/fpubh.2026.1995315 (PMC13738813; doi:10.3389/fpubh.2026.1995315)
Supplement: Supplementary file 1 [file Table_1.docx]

Table S1. Clinical characteristics of the 108 high-risk infants

| Patients | Sexes | Gestational ages (weeks) | Mode of delivery | Birth weight (g) | High-risk conditions | Feeding type within the first 6 months |
| --- | --- | --- | --- | --- | --- | --- |
| 1 | Male | 39^+6^ | Vaginal delivery | 3100 | Hyperbilirubinemia | Exclusive breastfeeding |
| 2 | Female | 38 | Vaginal delivery | 2260 | HDP | Mixed feeding |
| 3 | Male | >37 | Caesarean | 3480 | Hyperbilirubinemia | Mixed feeding |
| 4 | Female | 37 | Vaginal delivery | 4107 | Hyperbilirubinemia | Formula feeding |
| 5 | Male | 37 | Caesarean | 2390 | LBW | Mixed feeding |
| 6 | Male | 39 | Vaginal delivery | 4530 | Hyperbilirubinemia, GDM | Mixed feeding |
| 7 | Male | 37 | Caesarean | 2200 | Hyperbilirubinemia, Neonatal pneumonia, LBW, GDM, HDP | Formula feeding |
| 8 | Female | 38 | Vaginal delivery | 2290 | Hyperbilirubinemia, LBW | Exclusive breastfeeding |
| 9 | Male | >37 | NA | 2250 | LBW | Mixed feeding |
| 10 | Male | >37 | Vaginal delivery | 2400 | Fetal distress | Formula feeding |
| 11 | Male | 38 | NA | 3400 | HDP, AMA | Formula feeding |
| 12 | Male | >37 | NA | NA | Hyperbilirubinemia | Formula feeding |
| 13 | Female | >37 | Caesarean | 3950 | Hyperbilirubinemia | Exclusive breastfeeding |
| 14 | Female | 37 | Caesarean | 2900 | Maternal hypothyroidism, GDM | Formula feeding |
| 15 | Female | >37 | NA | 3100 | Fetal distress | Exclusive breastfeeding |
| 16 | Female | >37 | Vaginal delivery | 3050 | HDP | Mixed feeding |
| 17 | Male | 37 | Vaginal delivery | 2650 | Hyperbilirubinemia, Neonatal hypoglycemia | Exclusive breastfeeding |
| 18 | Male | 38^+2^ | Vaginal delivery | 3370 | GDM, HDP | Exclusive breastfeeding |
| 19 | Male | 39 | Caesarean | 3800 | Fetal distress | Mixed feeding |
| 20 | Male | 38 | Caesarean | 2670 | GDM | Formula feeding |
| 21 | Female | >37 | Caesarean | 3900 | GDM | Mixed feeding |
| 22 | Male | >37 | Caesarean | 3440 | Neonatal pneumonia | Formula feeding |
| 23 | Female | 39^+4^ | Vaginal delivery | 2980 | Fetal distress | Exclusive breastfeeding |
| 24 | Male | 38 | NA | 2350 | LBW | Mixed feeding |
| 25 | Male | >37 | NA | 3230 | GDM, HDP | Mixed feeding |
| 26 | Female | >37 | Caesarean | 2700 | GDM, HDP | Formula feeding |
| 27 | Female | 38^+6^ | Vaginal delivery | 2810 | Neonatal hypoglycemia | Mixed feeding |
| 28 | Male | >37 | Caesarean | 3170 | AMA | Formula feeding |
| 29 | Female | >37 | Caesarean | 2770 | AMA | Formula feeding |
| 30 | Male | >37 | NA | 3580 | GDM | Formula feeding |
| 31 | Male | 40^+1^ | Caesarean | 3750 | GDM, HDP | Exclusive breastfeeding |
| 32 | Female | 37^+6^ | Caesarean | 3020 | Hyperbilirubinemia | Formula feeding |
| 33 | Female | >37 | Caesarean | 3120 | Fetal distress | Mixed feeding |
| 34 | Male | 39^+3^ | Caesarean | 3250 | GDM | Mixed feeding |
| 35 | Male | >37 | Caesarean | 2800 | GDM | Mixed feeding |
| 36 | Male | >37 | Caesarean | 4050 | GDM | Exclusive breastfeeding |
| 37 | Female | >37 | NA | 3370 | Fetal distress | Mixed feeding |
| 38 | Female | >37 | Caesarean | 2400 | LBW | Formula feeding |
| 39 | Male | >37 | NA | 3400 | AMA | Mixed feeding |
| 40 | Female | 38^+6^ | Caesarean | 3580 | GDM | Exclusive breastfeeding |
| 41 | Female | 40 | Vaginal delivery | 3720 | GDM | Exclusive breastfeeding |
| 42 | Male | >37 | Vaginal delivery | 3300 | GDM | Mixed feeding |
| 43 | Female | 38^+2^ | Vaginal delivery | 3350 | Hyperbilirubinemia | Mixed feeding |
| 44 | Male | >37 | Vaginal delivery | 2850 | HDP | Mixed feeding |
| 45 | Female | >37 | NA | 2980 | AMA | Mixed feeding |
| 46 | Male | >37 | Caesarean | 3500 | GDM | Exclusive breastfeeding |
| 47 | Female | >37 | NA | 3900 | GDM | Mixed feeding |
| 48 | Female | 40^+1^ | Vaginal delivery | 4160 | Hyperbilirubinemia | Formula feeding |
| 49 | Male | >37 | Vaginal delivery | 3980 | GDM | Exclusive breastfeeding |
| 50 | Female | >37 | Caesarean | 2300 | LBW | Mixed feeding |
| 51 | Male | >37 | Vaginal delivery | 3950 | GDM | Mixed feeding |
| 52 | Female | >37 | NA | NA | GDM, AMA | Formula feeding |
| 53 | Male | >37 | NA | 2200 | LBW | Formula feeding |
| 54 | Female | 39^+3^ | Vaginal delivery | 3390 | Neonatal pneumonia, Neonatal sepsis, Fetal distress | Exclusive breastfeeding |
| 55 | Female | >37 | Vaginal delivery | 3680 | GDM | Mixed feeding |
| 56 | Female | 37 | Vaginal delivery | 3200 | GDM, HDP, AMA | Formula feeding |
| 57 | Female | 38 | Caesarean | 3220 | HDP | Formula feeding |
| 58 | Male | >37 | Caesarean | 4540 | GDM | Formula feeding |
| 59 | Male | >37 | NA | 3470 | GDM, HDP | Exclusive breastfeeding |
| 60 | Male | 39 | Caesarean | 3450 | Hyperbilirubinemia, Neonatal hypoglycemia | Mixed feeding |
| 61 | Female | >37 | Vaginal delivery | 3770 | AMA | Exclusive breastfeeding |
| 62 | Male | >37 | NA | 3400 | GDM | Mixed feeding |
| 63 | Male | 39 | Vaginal delivery | 3530 | HDP | Exclusive breastfeeding |
| 64 | Female | >37 | Caesarean | 2910 | HDP | Mixed feeding |
| 65 | Female | 38 | Vaginal delivery | 3540 | GDM | Mixed feeding |
| 66 | Female | 38 | Caesarean | 3550 | GDM | Exclusive breastfeeding |
| 67 | Male | 37 | Caesarean | 3070 | GDM | Formula feeding |
| 68 | Male | >37 | NA | 3160 | GDM | Mixed feeding |
| 69 | Male | 40 | Vaginal delivery | 3270 | GDM | Mixed feeding |
| 70 | Female | 37 | Vaginal delivery | 2400 | LBW | Mixed feeding |
| 71 | Male | >37 | NA | 3280 | AMA | Exclusive breastfeeding |
| 72 | Male | 39^+5^ | Caesarean | 2100 | LBW | Formula feeding |
| 73 | Male | >37 | Caesarean | 3750 | GDM, HDP | Exclusive breastfeeding |
| 74 | Male | >37 | NA | 3730 | GDM | Exclusive breastfeeding |
| 75 | Female | >37 | Vaginal delivery | 2200 | LBW | Mixed feeding |
| 76 | Female | 39 | Caesarean | 3750 | GDM | Mixed feeding |
| 77 | Female | 38 | Vaginal delivery | 3000 | Hyperbilirubinemia | Mixed feeding |
| 78 | Male | >37 | NA | 3000 | HDP | Mixed feeding |
| 79 | Female | >37 | NA | 4120 | GDM | Mixed feeding |
| 80 | Male | >37 | NA | 3570 | Fetal distress | Exclusive breastfeeding |
| 81 | Female | >37 | NA | 3470 | GDM | Formula feeding |
| 82 | Female | >37 | Vaginal delivery | 3060 | GDM | Mixed feeding |
| 83 | Female | 37 | Vaginal delivery | 3040 | AMA | Exclusive breastfeeding |
| 84 | Male | >37 | Vaginal delivery | 3340 | GDM | Formula feeding |
| 85 | Male | 38 | Vaginal delivery | 3600 | Maternal hypothyroidism | Exclusive breastfeeding |
| 86 | Female | >37 | Vaginal delivery | 3110 | GDM | Exclusive breastfeeding |
| 87 | Male | >37 | Caesarean | 3200 | GDM | Formula feeding |
| 88 | Female | 37 | Vaginal delivery | 2600 | Maternal hypothyroidism, GDM | Mixed feeding |
| 89 | Female | >37 | Caesarean | 3580 | GDM | Mixed feeding |
| 90 | Male | >37 | Caesarean | 3150 | Hyperbilirubinemia | Mixed feeding |
| 91 | Male | >37 | Vaginal delivery | 3670 | Maternal hypothyroidism | Exclusive breastfeeding |
| 92 | Female | >37 | Caesarean | 3970 | HDP | Exclusive breastfeeding |
| 93 | Female | 37 | Caesarean | 2150 | LBW | Exclusive breastfeeding |
| 94 | Female | >37 | Vaginal delivery | 3000 | GDM, Maternal hypothyroidism | Formula feeding |
| 95 | Male | >37 | Caesarean | 3370 | GDM | Exclusive breastfeeding |
| 96 | Female | 38^+5^ | NA | 3680 | GDM, Maternal hypothyroidism | Exclusive breastfeeding |
| 97 | Male | 37^+3^ | Caesarean | 3020 | HDP | Mixed feeding |
| 98 | Male | >37 | NA | 3840 | GDM | Mixed feeding |
| 99 | Male | >37 | Caesarean | 3200 | GDM | Exclusive breastfeeding |
| 100 | Female | 38^+3^ | Caesarean | 3450 | GDM, HDP | Mixed feeding |
| 101 | Male | >37 | Vaginal delivery | 2700 | AMA | Formula feeding |
| 102 | Female | >37 | Vaginal delivery | 3650 | GDM | Mixed feeding |
| 103 | Female | 41 | Vaginal delivery | 3400 | Maternal hypothyroidism | Formula feeding |
| 104 | Female | >37 | Vaginal delivery | 2900 | Maternal hypothyroidism | Formula feeding |
| 105 | Female | >37 | NA | NA | GDM | Formula feeding |
| 106 | Female | >37 | Caesarean | 2970 | Fetal distress | Mixed feeding |
| 107 | Female | >37 | Vaginal delivery | 3630 | HDP | Exclusive breastfeeding |
| 108 | Male | >37 | NA | 3100 | GDM, AMA | Formula feeding |

Abbreviations: GDM: Gestational diabetes mellitus; HDP: Hypertensive disorders of pregnancy; LBW: Low birth weight; AMA: Advanced maternal age; NA: Data not accessible. Gestational age was reported as documented in the electronic medical records. The notation ">37" indicated that the exact gestational age was unavailable, although the infant was documented as having been born at term.

Table S2. Bonferroni-adjusted pairwise comparisons of WHO-standardized anthropometric Z scores among feeding groups at 6, 8, and 12 months

| Outcome | Age (months) | Pairwise comparison | Mean difference | Bonferroni-adjusted 95% CI | Bonferroni-adjusted P value |
| --- | --- | --- | --- | --- | --- |
| LAZ | 6 | Exclusive breastfeeding *vs* mixed feeding | 0.543 | 0.000-1.085 | 0.050 |
|  |  | Exclusive breastfeeding *vs* formula feeding | 0.747 | 0.156-1.339 | 0.008* |
|  |  | Mixed feeding *vs* formula feeding | 0.205 | -0.343-0.752 | 1.000 |
| WAZ | 6 | Exclusive breastfeeding *vs* mixed feeding | 0.570 | -0.035-1.174 | 0.071 |
|  |  | Exclusive breastfeeding *vs* formula feeding | 0.924 | 0.266-1.583 | 0.003* |
|  |  | Mixed feeding *vs* formula feeding | 0.354 | -0.256-0.964 | 0.482 |
| LAZ | 8 | Exclusive breastfeeding *vs* mixed feeding | 0.588 | -0.092-1.268 | 0.113 |
|  |  | Exclusive breastfeeding *vs* formula feeding | 0.643 | -0.055-1.341 | 0.081 |
|  |  | Mixed feeding *vs* formula feeding | 0.055 | -0.618-0.728 | 1.000 |
| WAZ | 8 | Exclusive breastfeeding *vs* mixed feeding | 0.616 | -0.083-1.315 | 0.103 |
|  |  | Exclusive breastfeeding *vs* formula feeding | 0.975 | 0.257-1.693 | 0.004* |
|  |  | Mixed feeding *vs* formula feeding | 0.359 | -0.333-1.051 | 0.624 |
| HCZ | 8 | Exclusive breastfeeding *vs* mixed feeding | 0.590 | -0.052-1.231 | 0.082 |
|  |  | Exclusive breastfeeding *vs* formula feeding | 0.752 | 0.093-1.410 | 0.020* |
|  |  | Mixed feeding *vs* formula feeding | 0.162 | -0.473-0.797 | 1.000 |
| WAZ | 12 | Exclusive breastfeeding *vs* mixed feeding | 0.412 | -0.163-0.987 | 0.252 |
|  |  | Exclusive breastfeeding *vs* formula feeding | 0.676 | 0.042-1.310 | 0.033* |
|  |  | Mixed feeding *vs* formula feeding | 0.264 | -0.327-0.856 | 0.839 |

Note: Pairwise comparisons were performed following one-way analysis of variance when the overall between-group test was statistically significant. Mean differences are expressed as the first-named feeding group minus the second-named feeding group. P values and 95% confidence intervals were adjusted for the three pairwise comparisons using the Bonferroni method. Positive mean differences indicate higher Z scores in the first-named feeding group. WAZ, weight-for-age Z score; LAZ, length-for-age Z score; HCZ, head circumference-for-age Z score.

Table S3. Anthropometric measurements of high-risk infants at 6 months of age

| Patients | Length (cm) | Weight (kg) | Head circumference (cm) | Length-for-age z score | Weight-for-age z score | Head circumference-for-age z score |
| --- | --- | --- | --- | --- | --- | --- |
| 1 | 67.2 | 8.05 | 42.7 | -0.21 | 0.13 | -0.52 |
| 2 | 63.2 | 6.20 | 40.4 | -1.17 | -1.38 | -1.38 |
| 3 | 66.2 | 8.55 | 44.3 | -0.65 | 0.69 | 1.44 |
| 4 | 69.2 | 8.60 | 44.5 | 1.57 | 1.36 | 2.36 |
| 5 | 66.8 | 7.10 | 40.8 | -0.42 | -1.03 | -2.07 |
| 6 | 69.8 | 8.50 | 44.8 | 0.88 | 0.55 | 1.20 |
| 7 | 67.7 | 7.75 | 42.8 | -0.36 | -0.44 | -0.43 |
| 8 | 63.5 | 8.02 | 41.5 | -0.92 | 0.81 | 0.03 |
| 9 | 65.2 | 7.02 | 42.0 | -1.31 | -1.22 | -1.09 |
| 10 | 67.7 | 7.83 | 42.5 | 0.20 | -0.02 | -0.05 |
| 11 | 68.6 | 8.15 | 43.2 | 0.08 | 0.03 | -0.11 |
| 12 | 70.5 | 8.98 | 44.0 | 1.28 | 1.10 | 0.55 |
| 13 | 69.3 | 8.78 | 41.9 | 1.37 | 1.40 | -0.23 |
| 14 | 68.1 | 8.99 | 43.0 | 1.13 | 1.75 | 1.20 |
| 15 | 68.1 | 8.45 | 42.3 | 0.66 | 0.99 | 0.08 |
| 16 | 67.5 | 8.50 | 44.5 | 0.63 | 1.16 | 1.77 |
| 17 | 69.6 | 8.38 | 42.1 | 0.81 | 0.44 | -1.01 |
| 18 | 68.5 | 10.80 | 44.3 | 0.45 | 2.89 | 1.44 |
| 19 | 71.5 | 9.63 | 44.3 | 1.73 | 1.74 | 0.79 |
| 20 | 68.5 | 8.35 | 42.5 | 0.43 | 0.48 | -0.05 |
| 21 | 67.8 | 8.15 | 41.0 | 0.81 | 0.84 | -0.92 |
| 22 | 67.8 | 7.40 | 43.7 | 0.05 | -0.65 | 0.30 |
| 23 | 68.6 | 7.00 | 42.2 | 1.11 | -0.43 | 0.00 |
| 24 | 65.0 | 6.60 | 41.1 | -1.23 | -1.67 | -1.83 |
| 25 | 67.6 | 9.40 | 43.7 | -0.02 | 1.56 | 0.30 |
| 26 | 67.0 | 7.03 | 41.6 | 0.30 | -0.45 | -0.46 |
| 27 | 68.8 | 8.78 | 41.4 | 1.32 | 1.48 | -0.61 |
| 28 | 69.5 | 8.90 | 42.3 | 0.89 | 1.07 | -0.21 |
| 29 | 69.3 | 7.50 | 40.2 | 1.61 | 0.25 | -0.98 |
| 30 | 67.5 | 8.35 | 44.3 | -0.43 | 0.25 | 0.79 |
| 31 | 69.9 | 8.44 | 43.1 | 1.16 | 0.62 | 0.45 |
| 32 | 63.8 | 7.40 | 43.5 | -0.88 | 0.10 | 1.00 |
| 33 | 66.5 | 8.85 | 43.0 | 0.47 | 1.65 | 1.20 |
| 34 | 67.5 | 7.65 | 42.9 | -0.02 | -0.31 | 0.28 |
| 35 | 70.2 | 9.40 | 43.0 | 1.61 | 1.81 | 0.37 |
| 36 | 70.3 | 9.20 | 45.2 | 1.24 | 1.36 | 1.53 |
| 37 | 68.6 | 7.30 | 43.0 | 1.21 | -0.03 | 0.61 |
| 38 | 63.0 | 6.27 | 41.5 | -1.19 | -1.24 | 0.03 |
| 39 | 67.4 | 9.25 | 43.3 | -0.21 | 1.35 | -0.03 |
| 40 | 68.3 | 8.10 | 44.0 | 1.22 | 0.90 | 1.98 |
| 41 | 67.7 | 7.55 | 42.0 | 0.84 | 0.26 | -0.15 |
| 42 | 70.0 | 9.60 | 44.1 | 1.13 | 1.77 | 1.28 |
| 43 | 68.8 | 9.80 | 42.0 | 1.29 | 2.36 | -0.15 |
| 44 | 65.2 | 8.62 | 44.1 | -0.97 | 0.86 | 1.28 |
| 45 | 65.2 | 7.35 | 43.5 | -0.62 | -0.17 | 1.00 |
| 46 | 69.2 | 8.70 | 43.5 | 0.70 | 0.83 | 0.14 |
| 47 | 71.5 | 8.80 | 42.6 | 1.91 | 1.19 | 0.31 |
| 48 | 69.5 | 9.15 | 43.0 | 1.53 | 1.77 | 0.61 |
| 49 | 68.6 | 8.70 | 45.0 | 0.37 | 0.79 | 1.37 |
| 50 | 65.5 | 7.72 | 41.3 | -0.11 | 0.46 | -0.69 |
| 51 | 69.6 | 8.80 | 43.4 | 0.74 | 0.85 | 0.06 |
| 52 | 68.5 | 7.30 | 42.3 | 1.07 | -0.08 | 0.08 |
| 53 | 64.8 | 7.38 | 42.2 | -1.30 | -0.65 | -0.30 |
| 54 | 70.7 | 8.30 | 43.6 | 2.08 | 0.99 | 1.08 |
| 55 | 71.6 | 9.10 | 44.8 | 2.53 | 1.76 | 2.00 |
| 56 | 67.6 | 8.80 | 42.3 | 0.82 | 1.52 | 0.08 |
| 57 | 70.7 | 9.30 | 43.7 | 2.23 | 1.99 | 1.74 |
| 58 | 71.6 | 9.80 | 45.0 | 1.89 | 1.98 | 2.02 |
| 59 | 70.0 | 10.70 | 45.5 | 1.10 | 2.78 | 1.78 |
| 60 | 65.8 | 7.20 | 44.0 | -0.79 | -0.84 | 1.19 |
| 61 | 69.0 | 8.12 | 43.0 | 1.48 | 0.89 | 1.20 |
| 62 | 73.0 | 9.30 | 44.5 | 2.32 | 1.36 | 0.96 |
| 63 | 70.7 | 10.53 | 44.5 | 1.25 | 2.52 | 0.96 |
| 64 | 66.2 | 7.40 | 42.7 | 0.17 | 0.09 | 0.38 |
| 65 | 69.5 | 9.59 | 43.2 | 1.56 | 2.16 | 0.77 |
| 66 | 71.7 | 9.35 | 42.5 | 2.52 | 1.95 | 0.23 |
| 67 | 68.0 | 10.00 | 44.5 | 0.17 | 2.14 | 0.96 |
| 68 | 70.5 | 9.00 | 43.0 | 1.31 | 1.14 | -0.27 |
| 69 | 67.0 | 7.40 | 44.2 | -0.25 | -0.61 | 1.36 |
| 70 | 65.2 | 6.87 | 41.5 | -0.15 | -0.44 | 0.03 |
| 71 | 71.7 | 9.50 | 44.6 | 1.87 | 1.64 | 1.04 |
| 72 | 63.6 | 6.40 | 41.0 | -1.74 | -1.85 | -1.29 |
| 73 | 70.4 | 9.60 | 43.5 | 1.26 | 1.74 | 0.14 |
| 74 | 68.0 | 9.40 | 43.9 | 0.45 | 1.73 | 1.11 |
| 75 | 64.5 | 6.85 | 41.5 | -0.59 | -0.55 | -0.54 |
| 76 | 66.7 | 7.60 | 43.7 | 0.39 | 0.31 | 1.15 |
| 77 | 65.4 | 6.00 | 40.5 | -0.29 | -1.70 | -1.30 |
| 78 | 70.0 | 9.00 | 44.6 | 0.93 | 1.05 | 1.04 |
| 79 | 69.3 | 9.80 | 43.2 | 1.47 | 2.33 | 0.77 |
| 80 | 70.5 | 8.24 | 43.2 | 0.94 | 0.12 | -0.11 |
| 81 | 66.1 | 7.49 | 42.3 | -0.01 | 0.12 | 0.08 |
| 82 | 67.8 | 6.96 | 43.0 | 0.88 | -0.41 | 0.61 |
| 83 | 68.5 | 7.55 | 43.2 | 1.19 | 0.26 | 0.77 |
| 84 | 66.2 | 7.30 | 41.8 | -0.70 | -0.77 | -1.25 |
| 85 | 68.3 | 9.88 | 43.7 | 0.21 | 1.97 | 0.30 |
| 86 | 67.1 | 7.52 | 42.4 | 0.55 | 0.21 | 0.15 |
| 87 | 69.0 | 7.72 | 41.5 | 0.58 | -0.28 | -1.50 |
| 88 | 67.5 | 8.41 | 42.6 | 0.56 | 1.03 | 0.31 |
| 89 | 67.5 | 7.43 | 41.2 | 0.80 | 0.16 | -0.20 |
| 90 | 69.5 | 8.40 | 43.0 | 0.89 | 0.53 | 0.37 |
| 91 | 71.5 | 9.40 | 43.8 | 1.93 | 1.63 | 1.03 |
| 92 | 70.0 | 8.65 | 42.6 | 2.15 | 1.53 | 0.89 |
| 93 | 63.6 | 6.45 | 39.2 | -0.88 | -0.98 | -1.76 |
| 94 | 64.1 | 7.00 | 42.6 | -0.75 | -0.36 | 0.31 |
| 95 | 73.1 | 10.80 | 44.4 | 2.57 | 2.88 | 1.53 |
| 96 | 69.2 | 8.97 | 43.4 | 1.62 | 1.73 | 1.51 |
| 97 | 72.9 | 11.69 | 43.8 | 2.40 | 3.63 | 0.38 |
| 98 | 70.3 | 8.40 | 42.3 | 1.02 | 0.39 | -0.84 |
| 99 | 67.6 | 9.16 | 43.0 | 0.03 | 1.35 | 0.37 |
| 100 | 66.4 | 8.76 | 42.3 | 0.26 | 1.47 | 0.08 |
| 101 | 69.8 | 7.29 | 43.0 | 1.03 | -0.76 | 0.37 |
| 102 | 67.7 | 8.55 | 43.0 | 0.93 | 1.33 | 1.20 |
| 103 | 66.5 | 7.48 | 42.5 | 0.19 | 0.12 | 0.23 |
| 104 | 65.5 | 8.30 | 40.8 | -0.25 | 0.97 | -1.07 |
| 105 | 68.3 | 7.67 | 40.1 | 0.84 | 0.25 | -1.61 |
| 106 | 65.5 | 7.31 | 40.7 | 0.001 | 0.08 | -0.59 |
| 107 | 72.4 | 10.39 | 44.5 | 2.83 | 2.80 | 1.77 |
| 108 | 66.5 | 7.30 | 41.5 | -0.56 | -0.78 | -1.50 |

Note: Length-for-age, weight-for-age, and head circumference-for-age z scores were calculated according to the WHO Child Growth Standards. A negative z score indicates that the measurement is below the median of the age- and sex-specific reference population, whereas a positive z score indicates that it is above the reference median.

Table S4. Anthropometric measurements of high-risk infants at 8 months of age

| Patients | Length (cm) | Weight  (kg) | Head circumference (cm) | Length-for-age z score | Weight-for-age z score | Head circumference-for-age z score |
| --- | --- | --- | --- | --- | --- | --- |
| 1 | 71.0 | 8.40 | 43.6 | 0.28 | -0.18 | -0.31 |
| 2 | 66.0 | 6.90 | 41.8 | -1.25 | -1.22 | -1.18 |
| 3 | 70.7 | 9.70 | 45.2 | 0.16 | 1.16 | 0.99 |
| 4 | 73.0 | 9.23 | 44.8 | 1.42 | 1.04 | 1.08 |
| 5 | 70.8 | 7.53 | 41.9 | -0.41 | -1.47 | -2.11 |
| 6 | 71.6 | 9.40 | 45.5 | 0.32 | 0.74 | 0.78 |
| 7 | 70.3 | 8.23 | 44.1 | -0.35 | -0.53 | -0.35 |
| 8 | 68.5 | 8.97 | 42.5 | -0.37 | 0.86 | -0.65 |
| 9 | 67.2 | 7.63 | 43.0 | -1.59 | -1.14 | -1.23 |
| 10 | 70.0 | 8.61 | 43.8 | -0.38 | -0.06 | -0.59 |
| 11 | 71.9 | 8.45 | 44.2 | 0.47 | -0.23 | -0.27 |
| 12 | 72.6 | 10.05 | 46.1 | 0.90 | 1.42 | 1.26 |
| 13 | 72.3 | 9.10 | 43.3 | 0.91 | 0.83 | -0.05 |
| 14 | 72.8 | 10.01 | 45.0 | 1.68 | 1.85 | 1.23 |
| 15 | NA | NA | NA | NA | NA | NA |
| 16 | NA | NA | NA | NA | NA | NA |
| 17 | NA | NA | NA | NA | NA | NA |
| 18 | 71.5 | 11.70 | 46.2 | 0.38 | 2.84 | 1.34 |
| 19 | 73.5 | 9.80 | 46.4 | 1.05 | 1.06 | 1.50 |
| 20 | 71.6 | 9.06 | 43.5 | 0.42 | 0.45 | -0.83 |
| 21 | 71.5 | 9.48 | 43.5 | 0.95 | 1.33 | 0.10 |
| 22 | 71.7 | 8.06 | 45.1 | 0.45 | -0.64 | 0.46 |
| 23 | NA | NA | NA | NA | NA | NA |
| 24 | 68.3 | 7.10 | 42.4 | -1.33 | -1.91 | -1.71 |
| 25 | 70.3 | 9.86 | 45.0 | -0.25 | 1.19 | 0.38 |
| 26 | 70.5 | 7.62 | 42.4 | 0.41 | -0.51 | -0.73 |
| 27 | 72.3 | 9.25 | 42.7 | 1.41 | 1.19 | -0.50 |
| 28 | 72.3 | 9.40 | 43.0 | 0.76 | 0.80 | -1.23 |
| 29 | 71.8 | 8.85 | 42.1 | 1.28 | 0.87 | -0.95 |
| 30 | 71.5 | 9.09 | 45.3 | 0.42 | 0.50 | 1.07 |
| 31 | 72.5 | 9.05 | 44.2 | 0.87 | 0.46 | 0.18 |
| 32 | 67.8 | 8.60 | 44.5 | -0.55 | 0.57 | 0.85 |
| 33 | 69.6 | 10.0 | 44.6 | 0.37 | 1.86 | 1.34 |
| 34 | 69.5 | 8.16 | 44.9 | -0.49 | -0.49 | 0.75 |
| 35 | NA | NA | NA | NA | NA | NA |
| 36 | 71.5 | 9.70 | 46.0 | 0.36 | 1.07 | 1.18 |
| 37 | NA | NA | NA | NA | NA | NA |
| 38 | 64.3 | 6.80 | 42.2 | -1.84 | -1.27 | -0.48 |
| 39 | 71.7 | 10.40 | 45.0 | 0.43 | 1.71 | 0.38 |
| 40 | 71.0 | 8.80 | 45.8 | 0.98 | 0.84 | 2.26 |
| 41 | NA | NA | NA | NA | NA | NA |
| 42 | NA | NA | NA | NA | NA | NA |
| 43 | 72.2 | 10.50 | 42.8 | 1.37 | 2.20 | -0.43 |
| 44 | 68.3 | 9.21 | 45.3 | -1.11 | 0.58 | 0.62 |
| 45 | 67.2 | 7.90 | 44.2 | -0.76 | -0.10 | 0.63 |
| 46 | 71.5 | 9.30 | 44.6 | 0.34 | 0.67 | 0.06 |
| 47 | NA | NA | NA | NA | NA | NA |
| 48 | NA | NA | NA | NA | NA | NA |
| 49 | 74.0 | 10.00 | 47.5 | 1.13 | 1.18 | 2.39 |
| 50 | 68.3 | 8.30 | 43.0 | -0.24 | 0.33 | -0.28 |
| 51 | NA | NA | NA | NA | NA | NA |
| 52 | 70.0 | 7.80 | 44.0 | 0.36 | -0.24 | 0.48 |
| 53 | 67.5 | 7.70 | 44.0 | -1.40 | -1.02 | 0.02 |
| 54 | 73.5 | 9.00 | 45.0 | 2.10 | 1.06 | 1.65 |
| 55 | NA | NA | NA | NA | NA | NA |
| 56 | 70.7 | 9.50 | 44.0 | 0.59 | 1.34 | 0.48 |
| 57 | 75.6 | 10.30 | 44.7 | 2.89 | 2.09 | 1.00 |
| 58 | NA | NA | NA | NA | NA | NA |
| 59 | 72.2 | 10.50 | 46.5 | 0.67 | 1.81 | 1.58 |
| 60 | NA | NA | NA | NA | NA | NA |
| 61 | NA | NA | NA | NA | NA | NA |
| 62 | 75.6 | 9.90 | 45.6 | 2.21 | 1.26 | 0.86 |
| 63 | 72.5 | 10.90 | 45.6 | 0.66 | 2.09 | 0.86 |
| 64 | 69.3 | 8.00 | 44.2 | 0.16 | 0.02 | 0.63 |
| 65 | NA | NA | NA | NA | NA | NA |
| 66 | 72.3 | 10.00 | 44.0 | 1.29 | 1.76 | 0.48 |
| 67 | 71.5 | 11.20 | 46.2 | 0.38 | 2.43 | 1.34 |
| 68 | 71.9 | 9.93 | 44.5 | 0.66 | 1.35 | 0.42 |
| 69 | NA | NA | NA | NA | NA | NA |
| 70 | NA | NA | NA | NA | NA | NA |
| 71 | 76.5 | 10.50 | 46.5 | 2.44 | 1.73 | 1.58 |
| 72 | 67.6 | 7.50 | 42.4 | -1.41 | -1.29 | -1.71 |
| 73 | 74.5 | 10.30 | 44.9 | 1.74 | 1.64 | 0.30 |
| 74 | 72.2 | 10.20 | 45.1 | 0.76 | 1.58 | 0.91 |
| 75 | 68.2 | 7.75 | 42.3 | -0.32 | -0.25 | -0.80 |
| 76 | 71.1 | 8.40 | 44.8 | 0.94 | 0.43 | 1.08 |
| 77 | NA | NA | NA | NA | NA | NA |
| 78 | 72.0 | 9.5 | 45.5 | 0.58 | 0.88 | 0.78 |
| 79 | NA | NA | NA | NA | NA | NA |
| 80 | 71.7 | 8.71 | 44.4 | 0.43 | 0.06 | -0.10 |
| 81 | NA | NA | NA | NA | NA | NA |
| 82 | 70.5 | 7.74 | 44.2 | 0.63 | -0.27 | 0.63 |
| 83 | 71.6 | 8.50 | 45.0 | 0.85 | 0.38 | 1.23 |
| 84 | NA | NA | NA | NA | NA | NA |
| 85 | 71.5 | 10.45 | 44.7 | 0.57 | 1.87 | 0.58 |
| 86 | 69.5 | 7.98 | 44.2 | 0.25 | 0.00 | 0.63 |
| 87 | 72.0 | 8.52 | 43.3 | 0.56 | -0.14 | -0.99 |
| 88 | NA | NA | NA | NA | NA | NA |
| 89 | 70.5 | 8.44 | 43.0 | 0.57 | 0.41 | -0.28 |
| 90 | 73.3 | 9.78 | 44.0 | 1.21 | 1.17 | -0.43 |
| 91 | 74.4 | 10.05 | 44.7 | 1.76 | 1.44 | 0.58 |
| 92 | 73.0 | 9.53 | 43.5 | 2.00 | 1.57 | 0.51 |
| 93 | 65.0 | 7.10 | 40.5 | -1.63 | -0.96 | -2.16 |
| 94 | 68.0 | 7.93 | 43.4 | -0.33 | -0.02 | 0.02 |
| 95 | 76.5 | 12.18 | 45.7 | 2.66 | 3.25 | 0.94 |
| 96 | 72.6 | 9.82 | 44.3 | 1.68 | 1.74 | 1.12 |
| 97 | 77.4 | 12.10 | 44.5 | 3.05 | 3.17 | -0.02 |
| 98 | NA | NA | NA | NA | NA | NA |
| 99 | NA | NA | NA | NA | NA | NA |
| 100 | NA | NA | NA | NA | NA | NA |
| 101 | 73.0 | 8.06 | 44.7 | 0.72 | -0.79 | 0.14 |
| 102 | 70.8 | 8.90 | 44.3 | 0.70 | 0.84 | 0.70 |
| 103 | 70.5 | 8.38 | 44.3 | 0.71 | 0.42 | 0.70 |
| 104 | NA | NA | NA | NA | NA | NA |
| 105 | 71.5 | 7.73 | 40.9 | 0.87 | -0.37 | -1.86 |
| 106 | 70.6 | 8.41 | 42.2 | 0.71 | 0.43 | -0.88 |
| 107 | NA | NA | NA | NA | NA | NA |
| 108 | 68.1 | 7.76 | 42.6 | -1.74 | -1.26 | -1.91 |

Note: Length-for-age, weight-for-age, and head circumference-for-age z scores were calculated according to the WHO Child Growth Standards. A negative z score indicates that the measurement is below the median of the age- and sex-specific reference population, whereas a positive z score indicates that it is above the reference median; NA: Data not accessible.

Table S5. Anthropometric measurements of high-risk infants at 12 months of age

| Patients | Length  (cm) | Weight (kg) | Head circumference (cm) | Length-for-age z score | Weight-for-age z score | Head circumference-for-age z score |
| --- | --- | --- | --- | --- | --- | --- |
| 1 | 75.3 | 9.57 | 44.6 | -0.02 | 0.00 | -0.91 |
| 2 | 71.5 | 8.00 | 43.6 | -1.11 | -0.98 | -0.95 |
| 3 | 76.5 | 10.78 | 46.7 | 0.34 | 1.03 | 0.74 |
| 4 | 77.0 | 10.09 | 46.3 | 1.31 | 1.03 | 1.27 |
| 5 | 74.2 | 8.42 | 43.7 | -0.77 | -1.29 | -1.84 |
| 6 | 78.2 | 10.80 | 47.0 | 1.02 | 1.03 | 0.73 |
| 7 | 76.0 | 9.26 | 46.8 | -0.09 | -0.46 | 0.57 |
| 8 | 74.3 | 9.81 | 43.9 | -0.06 | 0.67 | -0.73 |
| 9 | 72.4 | 8.62 | 45.7 | -1.51 | -1.08 | -0.28 |
| 10 | 74.7 | 9.43 | 45.6 | -0.40 | -0.19 | -0.12 |
| 11 | 76.5 | 9.75 | 45.8 | 0.27 | 0.08 | -0.21 |
| 12 | 77.0 | 10.43 | 47.1 | 0.45 | 0.68 | 0.80 |
| 13 | 75.2 | 10.10 | 44.0 | 0.32 | 0.92 | -0.66 |
| 14 | 77.1 | 10.57 | 46.3 | 1.22 | 1.34 | 1.27 |
| 15 | 76.5 | 9.63 | 45.3 | 0.33 | 0.33 | 0.09 |
| 16 | 77.0 | 9.75 | 47.3 | 1.05 | 0.65 | 1.77 |
| 17 | 78.1 | 10.73 | 45.1 | 0.94 | 0.96 | -0.75 |
| 18 | 75.5 | 11.84 | 47.3 | -0.02 | 1.93 | 1.21 |
| 19 | 79.5 | 11.05 | 47.0 | 1.41 | 1.18 | 0.73 |
| 20 | 76.3 | 9.76 | 45.5 | 0.14 | 0.06 | -0.44 |
| 21 | 77.0 | 10.62 | 44.3 | 1.02 | 1.31 | -0.44 |
| 22 | 76.9 | 9.30 | 47.2 | 0.26 | -0.43 | 0.88 |
| 23 | 75.0 | 8.30 | 44.6 | 0.34 | -0.64 | -0.22 |
| 24 | 72.0 | 8.60 | 44.5 | -1.68 | -1.10 | -1.22 |
| 25 | 76.0 | 10.38 | 45.8 | 0.09 | 0.67 | -0.21 |
| 26 | 76.0 | 8.64 | 44.3 | 0.55 | -0.38 | -0.44 |
| 27 | 77.0 | 10.10 | 44.2 | 1.15 | 0.97 | -0.51 |
| 28 | 79.1 | 10.90 | 45.6 | 1.38 | 1.11 | -0.36 |
| 29 | 78.7 | 10.30 | 44.7 | 1.82 | 1.13 | 0.09 |
| 30 | 77.3 | 10.50 | 48.0 | 0.46 | 0.69 | 1.51 |
| 31 | 78.4 | 10.25 | 46.7 | 1.07 | 0.54 | 0.49 |
| 32 | 73.6 | 10.35 | 47.0 | -0.46 | 1.04 | 1.55 |
| 33 | 75.3 | 11.30 | 46.7 | 0.31 | 1.78 | 1.33 |
| 34 | 76.0 | 9.41 | 47.2 | 0.06 | -0.25 | 0.88 |
| 35 | 79.0 | 11.10 | 45.6 | 1.46 | 1.33 | -0.12 |
| 36 | 75.8 | 10.30 | 46.7 | 0.03 | 0.60 | 0.74 |
| 37 | 77.1 | 8.90 | 45.8 | 1.14 | -0.07 | 0.66 |
| 38 | 69.3 | 7.25 | 42.8 | -1.80 | -1.74 | -1.32 |
| 39 | 75.9 | 11.05 | 46.0 | 0.05 | 1.24 | -0.05 |
| 40 | 75.3 | 9.70 | 47.4 | 0.57 | 0.68 | 2.08 |
| 41 | 74.1 | 8.70 | 44.3 | 0.01 | -0.24 | -0.44 |
| 42 | 76.0 | 9.10 | 45.8 | 0.19 | -0.50 | 0.03 |
| 43 | 77.0 | 11.62 | 44.3 | 1.16 | 2.08 | -0.21 |
| 44 | 73.5 | 10.02 | 47.0 | -0.96 | 0.34 | 0.73 |
| 45 | 73.0 | 8.13 | 44.7 | -0.54 | -0.86 | -0.14 |
| 46 | 74.8 | 9.78 | 45.8 | -0.52 | 0.07 | -0.21 |
| 47 | 76.6 | 10.09 | 44.5 | 0.75 | 0.86 | -0.29 |
| 48 | 79.0 | 11.60 | 46.5 | 1.89 | 2.04 | 1.18 |
| 49 | 74.7 | 10.20 | 48.3 | -0.39 | 0.53 | 1.99 |
| 50 | 73.5 | 9.20 | 44.1 | -0.32 | 0.17 | -0.59 |
| 51 | 79.0 | 11.06 | 46.9 | 1.22 | 1.19 | 0.65 |
| 52 | 73.4 | 8.22 | 44.0 | -0.49 | -0.81 | -0.66 |
| 53 | 74.0 | 8.32 | 45.4 | -0.76 | -1.36 | -0.52 |
| 54 | 78.0 | 9.70 | 45.0 | 1.60 | 0.67 | 0.31 |
| 55 | 79.2 | 11.00 | 47.0 | 1.95 | 1.62 | 1.55 |
| 56 | 74.5 | 10.20 | 45.3 | 0.18 | 1.05 | 0.30 |
| 57 | 81.6 | 11.20 | 46.6 | 2.86 | 1.76 | 1.25 |
| 58 | 78.5 | 11.59 | 47.6 | 1.14 | 1.68 | 1.19 |
| 59 | 77.0 | 11.40 | 48.0 | 0.19 | 1.39 | 1.51 |
| 60 | 71.0 | 7.80 | 46.5 | -1.98 | -1.92 | 0.58 |
| 61 | 75.5 | 9.70 | 47.0 | 0.74 | 0.72 | 1.79 |
| 62 | 80.8 | 10.80 | 46.8 | 2.01 | 0.99 | 0.57 |
| 63 | 76.5 | 11.38 | 46.6 | 0.40 | 1.56 | 0.66 |
| 64 | 72.2 | 8.70 | 45.3 | -0.72 | -0.23 | 0.30 |
| 65 | 75.3 | 10.20 | 46.0 | 0.55 | 1.08 | 1.05 |
| 66 | 78.5 | 12.10 | 46.0 | 1.60 | 2.33 | 0.81 |
| 67 | 75.5 | 11.10 | 47.0 | -0.08 | 1.30 | 0.97 |
| 68 | 77.5 | 10.90 | 45.3 | 0.78 | 1.14 | -0.36 |
| 69 | 76.6 | 9.00 | 46.5 | 0.41 | -0.61 | 0.58 |
| 70 | 73.6 | 8.43 | 45.6 | 0.13 | -0.36 | 0.75 |
| 71 | 78.6 | 11.70 | 47.6 | 1.20 | 1.78 | 1.44 |
| 72 | 71.0 | 7.40 | 43.5 | -2.05 | -2.43 | -2.00 |
| 73 | 79.5 | 11.00 | 46.7 | 1.58 | 1.21 | 0.74 |
| 74 | 77.0 | 10.90 | 46.5 | 0.60 | 1.15 | 0.58 |
| 75 | 70.7 | 8.88 | 44.3 | -1.33 | -0.08 | -0.44 |
| 76 | 77.2 | 9.70 | 47.0 | 1.13 | 0.61 | 1.55 |
| 77 | 73.0 | 7.60 | 43.5 | -0.45 | -1.38 | -1.03 |
| 78 | 76.3 | 10.70 | 47.4 | 0.17 | 0.92 | 1.04 |
| 79 | 78.0 | 10.30 | 45.7 | 1.40 | 1.07 | 0.59 |
| 80 | 76.2 | 9.53 | 45.9 | 0.11 | -0.15 | -0.13 |
| 81 | 71.1 | 9.10 | 45.2 | -1.17 | 0.12 | 0.22 |
| 82 | 75.5 | 8.24 | 45.5 | 0.56 | -0.68 | 0.44 |
| 83 | 76.0 | 9.40 | 45.5 | 0.43 | 0.26 | 0.44 |
| 84 | 74.0 | 8.31 | 44.0 | -0.73 | -1.36 | -1.38 |
| 85 | 74.9 | 12.11 | 46.7 | -0.53 | 2.02 | 0.49 |
| 86 | 73.1 | 8.77 | 45.5 | -0.38 | -0.18 | 0.44 |
| 87 | 76.6 | 9.82 | 45.5 | 0.07 | 0.04 | -0.44 |
| 88 | 76.8 | 9.92 | 45.4 | 1.05 | 0.82 | 0.37 |
| 89 | NA | NA | NA | NA | NA | NA |
| 90 | 78.5 | 10.45 | 45.8 | 1.16 | 0.73 | 0.03 |
| 91 | 79.1 | 11.37 | 47.0 | 1.36 | 1.49 | 0.73 |
| 92 | 78.4 | 10.60 | 45.2 | 1.91 | 1.44 | 0.45 |
| 93 | 70.3 | 7.80 | 42.2 | -1.25 | -1.04 | -1.76 |
| 94 | NA | NA | NA | NA | NA | NA |
| 95 | 81.3 | 13.35 | 47.3 | 2.43 | 3.05 | 1.21 |
| 96 | 76.7 | 10.37 | 45.9 | 0.90 | 1.13 | 0.74 |
| 97 | 78 | 13.5 | 47.5 | 0.97 | 3.12 | 1.37 |
| 98 | 77.5 | 10.03 | 44.7 | 0.81 | 0.39 | -0.83 |
| 99 | 74.8 | 9.80 | 45.1 | -0.88 | -0.07 | -0.96 |
| 100 | 76.2 | 10.00 | 45.0 | 0.49 | 0.75 | 0.08 |
| 101 | 76.8 | 9.14 | 46.0 | 0.20 | -0.60 | -0.05 |
| 102 | 74.5 | 10.17 | 46.2 | 0.16 | 1.02 | 0.96 |
| 103 | 73.0 | 8.80 | 45.5 | -0.48 | -0.17 | 0.44 |
| 104 | 73.6 | 9.00 | 43.0 | -0.23 | 0.02 | -1.40 |
| 105 | 76.0 | 8.57 | 42.4 | 0.52 | -0.46 | -1.84 |
| 106 | 75.9 | 9.15 | 43.9 | 0.69 | 0.16 | -0.73 |
| 107 | 79.8 | 11.40 | 48.0 | 1.95 | 1.81 | 2.28 |
| 108 | NA | NA | NA | NA | NA | NA |

Note: Length-for-age, weight-for-age, and head circumference-for-age z scores were calculated according to the WHO Child Growth Standards. A negative z score indicates that the measurement is below the median of the age- and sex-specific reference population, whereas a positive z score indicates that it is above the reference median; NA: Data not accessible.

Table S6. Developmental Screening Test (DST) scores among term high-risk infants according to feeding pattern

| Patients | Assessment age (months) | Gross motor raw score | Gross motor developmental age (months) | Social adaptation raw score | Social adaptation developmental age (months) | Intelligence raw score | Intelligence developmental age (months) | Developmental quotient (DQ) | Mental index (MI) |
| --- | --- | --- | --- | --- | --- | --- | --- | --- | --- |
| 1 | 12 | 10 | 8 | 14 | 12 | 25 | 11 | 86 | 92 |
| 2 | 12 | 12 | 10 | 14 | 12 | 25 | 11 | 79 | 79 |
| 3 | - | - | - | - | - | - | - | - | - |
| 4 | - | - | - | - | - | - | - | - | - |
| 5 | 8 | 11 | 9 | 9 | 7 | 19 | 8 | 89 | 85 |
| 6 | - | - | - | - | - | - | - | - | - |
| 7 | 8 | 9 | 7 | 9 | 7 | 19 | 8 | 78 | 85 |
| 8 | 8 | 9 | 7 | 9 | 7 | 19 | 8 | 78 | 85 |
| 9 | 12 | 11 | 9 | 12 | 10 | 26 | 11 | 71 | 86 |
| 10 | 12 | 12 | 10 | 14 | 12 | 25 | 11 | 96 | 92 |
| 11 | 8 | 9 | 7 | 7 | 5 | 20 | 8 | 72 | 94 |
| 12 | 12 | 12 | 10 | 13 | 11 | 25 | 11 | 91 | 92 |
| 13 | - | - | - | - | - | - | - | - | - |
| 14 | 12 | 13 | 11 | 13 | 11 | 26 | 11 | 83 | 86 |
| 15 | 12 | 14 | 12 | 14 | 12 | 26 | 11 | 91 | 86 |
| 16 | 12 | 14 | 12 | 14 | 12 | 28 | 12 | 99 | 100 |
| 17 | - | - | - | - | - | - | - | - | - |
| 18 | 8 | 9 | 7 | 10 | 8 | 19 | 8 | 83 | 85 |
| 19 | 12 | 13 | 11 | 13 | 11 | 26 | 11 | 83 | 86 |
| 20 | 8 | 10 | 8 | 9 | 7 | 20 | 8 | 89 | 94 |
| 21 | 12 | 12 | 10 | 13 | 11 | 25 | 11 | 75 | 79 |
| 22 | 12 | 14 | 12 | 13 | 11 | 26 | 11 | 87 | 86 |
| 23 | 12 | 13 | 11 | 13 | 11 | 26 | 11 | 83 | 86 |
| 24 | 8 | 10 | 8 | 9 | 7 | 19 | 8 | 83 | 85 |
| 25 | 8 | 11 | 9 | 9 | 7 | 18 | 7 | 83 | 76 |
| 26 | 8 | 9 | 7 | 9 | 7 | 19 | 8 | 78 | 85 |
| 27 | 8 | 11 | 9 | 9 | 7 | 19 | 8 | 89 | 85 |
| 28 | - | - | - | - | - | - | - | - | - |
| 29 | 8 | 11 | 9 | 9 | 7 | 19 | 8 | 89 | 85 |
| 30 | 8 | 9 | 7 | 8 | 6 | 18 | 7 | 66 | 76 |
| 31 | - | - | - | - | - | - | - | - | - |
| 32 | 12 | 13 | 11 | 12 | 10 | 25 | 11 | 75 | 79 |
| 33 | 8 | 9 | 7 | 9 | 7 | 20 | 8 | 83 | 94 |
| 34 | - | - | - | - | - | - | - | - | - |
| 35 | 12 | 12 | 10 | 14 | 12 | 27 | 12 | 106 | 111 |
| 36 | 12 | 11 | 9 | 14 | 12 | 25 | 11 | 75 | 79 |
| 37 | 12 | 11 | 9 | 14 | 12 | 26 | 11 | 79 | 86 |
| 38 | 8 | 9 | 7 | 9 | 7 | 18 | 7 | 72 | 76 |
| 39 | 8 | 9 | 7 | 9 | 7 | 18 | 7 | 72 | 76 |
| 40 | - | - | - | - | - | - | - | - | - |
| 41 | 12 | 13 | 11 | 14 | 12 | 28 | 12 | 95 | 100 |
| 42 | - | - | - | - | - | - | - | - | - |
| 43 | 8 | 9 | 7 | 9 | 7 | 19 | 8 | 78 | 85 |
| 44 | 8 | 9 | 7 | 8 | 6 | 18 | 7 | 66 | 76 |
| 45 | 12 | 14 | 12 | 13 | 11 | 26 | 11 | 87 | 86 |
| 46 | 8 | 10 | 8 | 10 | 8 | 21 | 9 | 100 | 103 |
| 47 | 12 | 12 | 10 | 14 | 12 | 26 | 11 | 83 | 86 |
| 48 | 12 | 13 | 11 | 14 | 12 | 27 | 12 | 91 | 93 |
| 49 | 12 | 10 | 8 | 13 | 11 | 20 | 8 | 56 | 46 |
| 50 | 12 | 13 | 11 | 14 | 12 | 27 | 12 | 91 | 93 |
| 51 | - | - | - | - | - | - | - | - | - |
| 52 | 8 | 9 | 7 | 9 | 7 | 19 | 8 | 78 | 85 |
| 53 | 12 | 13 | 11 | 14 | 12 | 25 | 11 | 83 | 79 |
| 54 | - | - | - | - | - | - | - | - | - |
| 55 | 12 | 13 | 11 | 13 | 11 | 27 | 12 | 87 | 93 |
| 56 | - | - | - | - | - | - | - | - | - |
| 57 | - | - | - | - | - | - | - | - | - |
| 58 | - | - | - | - | - | - | - | - | - |
| 59 | 8 | 9 | 7 | 9 | 7 | 18 | 7 | 72 | 76 |
| 60 | - | - | - | - | - | - | - | - | - |
| 61 | - | - | - | - | - | - | - | - | - |
| 62 | 8 | 10 | 8 | 9 | 7 | 21 | 9 | 95 | 103 |
| 63 | 12 | 14 | 12 | 14 | 12 | 27 | 12 | 116 | 111 |
| 64 | 8 | 9 | 7 | 9 | 7 | 19 | 8 | 78 | 85 |
| 65 | - | - | - | - | - | - | - | - | - |
| 66 | 8 | 11 | 9 | 9 | 7 | 19 | 8 | 89 | 85 |
| 67 | 8 | 8 | 6 | 8 | 6 | 15 | 6 | 62 | 50 |
| 68 | 12 | 12 | 10 | 13 | 11 | 26 | 11 | 96 | 102 |
| 69 | 12 | 13 | 11 | 13 | 11 | 27 | 12 | 106 | 111 |
| 70 | - | - | - | - | - | - | - | - | - |
| 71 | 12 | 13 | 11 | 13 | 11 | 26 | 11 | 83 | 86 |
| 72 | 12 | 10 | 8 | 13 | 11 | 27 | 12 | 75 | 93 |
| 73 | 12 | 14 | 12 | 13 | 11 | 26 | 11 | 87 | 86 |
| 74 | - | - | - | - | - | - | - | - | - |
| 75 | 8 | 9 | 7 | 9 | 7 | 19 | 8 | 78 | 85 |
| 76 | 8 | 9 | 7 | 9 | 7 | 16 | 6 | 61 | 58 |
| 77 | 12 | 13 | 11 | 13 | 11 | 25 | 11 | 79 | 79 |
| 78 | 12 | 13 | 11 | 13 | 11 | 25 | 11 | 79 | 79 |
| 79 | 12 | 14 | 12 | 14 | 12 | 25 | 11 | 87 | 79 |
| 80 | 8 | 11 | 9 | 10 | 8 | 21 | 9 | 106 | 103 |
| 81 | 12 | 12 | 10 | 13 | 11 | 26 | 11 | 79 | 86 |
| 82 | 8 | 9 | 7 | 9 | 7 | 20 | 8 | 83 | 94 |
| 83 | 12 | 14 | 12 | 14 | 12 | 29 | 15 | 103 | 108 |
| 84 | - | - | - | - | - | - | - | - | - |
| 85 | 8 | 9 | 7 | 9 | 7 | 19 | 8 | 95 | 101 |
| 86 | 12 | 13 | 11 | 14 | 12 | 26 | 11 | 87 | 86 |
| 87 | 8 | 11 | 9 | 9 | 7 | 19 | 8 | 89 | 85 |
| 88 | 8 | 14 | 12 | 13 | 11 | 26 | 11 | 87 | 86 |
| 89 | - | - | - | - | - | - | - | - | - |
| 90 | 8 | 11 | 9 | 9 | 7 | 18 | 7 | 83 | 76 |
| 91 | 12 | 12 | 10 | 14 | 12 | 26 | 11 | 83 | 86 |
| 92 | 8 | 9 | 7 | 10 | 8 | 18 | 7 | 78 | 76 |
| 93 | - | - | - | - | - | - | - | - | - |
| 94 | 8 | 9 | 7 | 9 | 7 | 19 | 8 | 78 | 85 |
| 95 | - | - | - | - | - | - | - | - | - |
| 96 | 8 | 10 | 8 | 9 | 7 | 20 | 8 | 89 | 94 |
| 97 | 12 | 12 | 10 | 13 | 11 | 26 | 11 | 96 | 102 |
| 98 | - | - | - | - | - | - | - | - | - |
| 99 | - | - | - | - | - | - | - | - | - |
| 100 | - | - | - | - | - | - | - | - | - |
| 101 | 8 | 10 | 8 | 9 | 7 | 18 | 7 | 78 | 76 |
| 102 | 8 | 9 | 7 | 9 | 7 | 20 | 8 | 83 | 94 |
| 103 | 8 | 11 | 9 | 11 | 9 | 21 | 9 | 128 | 118 |
| 104 | 12 | 14 | 12 | 14 | 12 | 26 | 11 | 91 | 86 |
| 105 | 8 | 9 | 7 | 9 | 7 | 20 | 8 | 83 | 94 |
| 106 | 8 | 14 | 12 | 14 | 12 | 26 | 11 | 91 | 86 |
| 107 | 12 | 15 | 15 | 14 | 12 | 26 | 11 | 95 | 86 |
| 108 | 8 | 12 | 10 | 9 | 7 | 22 | 9 | 92 | 98 |

Note: According to the DST criteria, DQ ≥85 indicates normal development, 70-84 indicates suspected developmental delay, and <70 indicates developmental delay. As all participants were younger than 3 years, neurodevelopment was evaluated using DQ only; -: Not performed.
